# Diagnosis, Management and Theragnostic Approach of Gastro-Entero-Pancreatic Neuroendocrine Neoplasms

**DOI:** 10.3390/cancers15133483

**Published:** 2023-07-04

**Authors:** Leandra Piscopo, Emilia Zampella, Sara Pellegrino, Fabio Volpe, Carmela Nappi, Valeria Gaudieri, Rosa Fonti, Silvana Del Vecchio, Alberto Cuocolo, Michele Klain

**Affiliations:** Department of Advanced Biomedical Sciences, University of Naples, Federico II, 80131 Naples, Italy; leandra.piscopo@unina.it (L.P.); emilia.zampella@unina.it (E.Z.); sara.pellegrino@unina.it (S.P.); fabio.volpe@unina.it (F.V.); c.nappi@unina.it (C.N.); valeria.gaudieri@unina.it (V.G.); rosa.fonti@unina.it (R.F.); delvecc@unina.it (S.D.V.); cuocolo@unina.it (A.C.)

**Keywords:** neuroendocrine neoplasms, somatostatin receptors, Gallium^68^, ^18^F-FDG, SPECT, PET/CT, peptide receptor radionuclide therapy, theragnosis

## Abstract

**Simple Summary:**

Gastro-entero-pancreatic neuroendocrine neoplasms (GEP-NENs) are the most common neuroendocrine tumors, with the ability of overexpressing somatostatin receptors (SSTRs) on the cells’ surface. The prevalence and the incidence of GEP-NENs have been constantly increasing over the last years, reflecting the improved sensitivity of imaging modalities and effectiveness of new therapeutic options. The theragnostic approach, which is able to combine diagnosis and therapy, is widely applied in GEP-NENs patients through the administration of radiolabeled SSTR analogs. In the era of personalized medicine, this combined approach represents an opportunity to plan a real tailored treatment in patients with GEP-NENs.

**Abstract:**

Gastro-entero-pancreatic neuroendocrine neoplasms (GEP-NENs) constitute an ideal target for radiolabeled somatostatin analogs. The theragnostic approach is able to combine diagnosis and therapy by the identification of a molecular target that can be diagnosed and treated with the same radiolabeled compound. During the last years, advances in functional imaging with the introduction of somatostatin analogs and peptide receptor radionuclide therapy, have improved the diagnosis and treatment of GEP-NENs. Moreover, PET/CT imaging with ^18^F-FDG represents a complementary tool for prognostic evaluation of patients with GEP-NENs. In the field of personalized medicine, the theragnostic approach has emerged as a promising tool in diagnosis and management of patients with GEP-NENs. The aim of this review is to summarize the current evidence on diagnosis and management of patients with GEP-NENs, focusing on the theragnostic approach.

## 1. Introduction

Neuroendocrine neoplasms (NENs) constitute a heterogeneous group of malignancies arising from the diffuse neuroendocrine cell system. Gastro-entero-pancreatic (GEP) NENs account for more than 70% of neuroendocrine neoplasms [1,2]. They share the ability of overexpressing somatostatin receptors (SSTRs) on the cells’ surface, in particular subtypes 2 and 5 [3,4]. The GEP-NENs may occur as asymptomatic or associated with specific syndromes of uncontrolled hormone hypersecretion, showing a very complex and heterogeneous clinical behavior. The GEP-NENs range from well-differentiated neuroendocrine tumors (NETs) to neuroendocrine carcinomas (NECs), and tumor aggressiveness and prognosis are strongly related to the degree of tumor differentiation [2,5]. The 2022 WHO Classification stratifies GEP-NENs into a 3-tiered grading system, based on mitotic rate and Ki-67 labeling index; low-grade tumors are classified as grade 1 (G1), intermediate-grades as grade 2 (G2) and high-grades as grade 3 (G3), respectively [6]. Among G3 tumors, those presenting a number of mitoses or a Ki-67 index higher than 20% and a more well-differentiated morphology than NET carcinomas have been identified as well-differentiated G3 NET [6]. The GEP-NENs show a low proliferation rate and a low tendency to metastasize. However, the vast majority of patients show distant metastases at diagnosis, due to the absence of hormone-associated symptoms, leading to late diagnosis and subsequent delays in treatment that can result in a tumor’s metastatic progression [7]. In contrast, GEP neuroendocrine carcinomas (NECs) are inevitably and uniformly poorly differentiated; they often progress rapidly and are accompanied by multiple synchronous distant metastases upon diagnosis, leading to a poor prognosis [7]. Although GEP-NENs are considered relatively rare tumors, both prevalence (1.0–1.5% of all GEP neoplasms) and incidence rates (6.98 and 0.4 new cases per year per 100,000 individuals in the United States for GEP-NETs and GEP-NECs, respectively) have been constantly increasing over the last three decades [8]. This evidence reflects the improved sensitivity of available imaging modalities and the effectiveness of the new therapeutic options. The SSTRs overexpression in GEP-NENs constitutes an ideal target for diagnosis and therapy with radiolabeled somatostatin analogs. In this context, nuclear medicine techniques, such as Positron Emission Tomography (PET) and Single Photon Emission Computed Tomography (SPECT), have been proposed as crucial tools in the management of GEP-NENs.

The theragnostic approach is able to combine diagnosis and therapy by using the same molecule for the identification of a therapeutic target that can be treated with the same compound [9]. Peptide receptor radionuclide therapy (PRRT) has been validated as an effective systemic treatment of patients with NENs and high somatostatin receptor expression. In the field of personalized medicine, the theragnostic has emerged as a promising tool in diagnosis and management of GEP-NENs. The aim of this review is to summarize the current evidence on diagnosis and management of patients with GEP-NENs, focusing on the theragnostic approach and elucidating some points still worthy of debate.

## 2. Nuclear Theragnostic

Nuclear medicine is based on the ability of different radiotracers to reproduce biological, cellular or metabolic pathways, in order to evaluate specific mechanisms of disease [10]. For this purpose, radioactive isotopes can be used alone or combined with different molecules.

**Figure 1:**
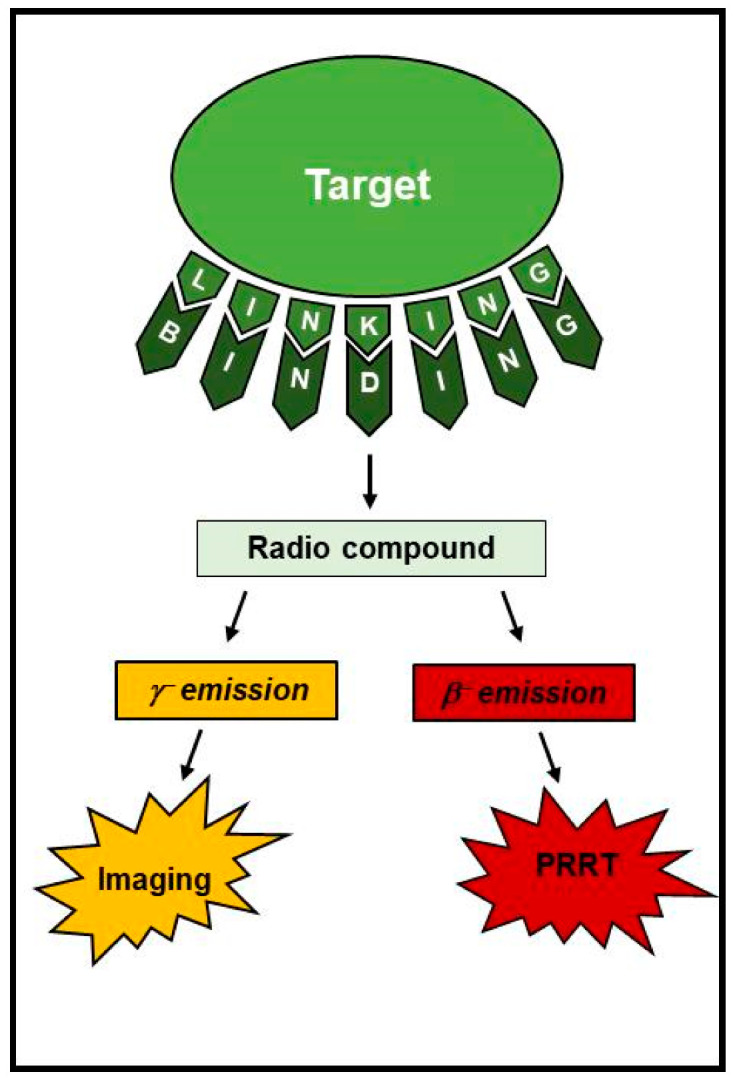
The theragnostic approach.

Radioisotopes may have both diagnostic and therapeutic potential, and each application is related to their decay properties. In particular, γ-emitters are used for diagnostic imaging by using SPECT or PET. Moreover, the radionuclides emitting low-range highly ionizing radiation, such as α- and β-emitters, are widely used for therapeutic purposes. The theragnostic approach has emerged as a fundamental and interdisciplinary junction between diagnostic and therapeutic applications of nuclear imaging (Figure 1) [11,12]. Theragnostic agents are based on the administration of disease-related biomarkers labeled with radioisotopes that can be visualized by molecular imaging techniques, allowing characterization of the diseased target tissue and providing imaging readouts of therapy response. According to emission properties of different radioisotopes, the same compound can be used for therapeutic purposes, leading to a real personalized beneficial approach. The applications of a specific isotope depend on its particle emission: Radioisotopes able to emit both γ and β radiation are able to provide both diagnostic and therapeutic effects. In this context, the more widely used theragnostic agent is Iodine-131 (^131^I), which is used for diagnosis and treatment of patients with differentiated thyroid cancer [13,14,15]. Radioiodine-based diagnosis, evaluation and therapy for differentiated thyroid cancer was the first successful theragnostic system, and it is the best example of a classical procedure that has maintained high clinical relevance in modern medicine [16]. However, in the clinical practice, the use of double emitters may lead to unnecessarily high radiation exposure and poor image quality when they are administered for diagnostic purposes only. Therefore, theragnostic pairs, with similar structures and a matching pair of radionuclides, had better serve diagnostic purposes by lowering radiation burden and achieving better image quality, differentiating the purely diagnostic from the therapy-tracking radioisotope. Continuous innovations in molecular biology, radiochemistry and hybrid imaging methods have led to major developments and improvements in diagnostic accuracy and to the availability of cutting-edge theragnostic procedures.

## 3. The Somatostatin Signaling

Somatostatin is a cyclic peptide that exerts inhibitory effects on both the secretion of endocrine and exocrine hormones [17,18]. The greater amount of the circulating somatostatin is produced by gastrointestinal system; in particular, more than 65% is secreted by delta cells of the islets of Langerhans, where it regulates both insulins and glucagon release from adjacent cells and the 5% by pancreatic beta cells [19,20].

The somatostatin activity is mediated by its binding to five subtypes of SSTR that belong to the superfamily of G protein-coupled receptors (GPCRs), characterized by a core of seven transmembrane helices [21]. The SSTRs inhibit cell proliferation and signaling molecules by inhibition of adenylate cyclase and reduction of intracellular calcium [22]. 

The SSTRs are expressed in many tissues, and their effects under physiological conditions are partially determined by the subtypes expressed on the tissue surface [23]. However, the SSTRs’ expression has also been reported in several tumors; in particular, SSTR2 is common in gliomas, medulloblastomas, paragangliomas and neuroblastomas [24]. The SSTR expression levels have been reported in most GEP-NENs [25,26]. Since SSTRs are found on the surface of tumor cells, they have the potential to serve as diagnostic markers and be used for receptor treatment.

## 4. Functional Imaging by SST Analogs

In 1989 Krenning and Co. [27] first introduced in vivo imaging of STRRs expression by using ^111^In-pentetreotide, a radiolabeled compound commercially available as OctreoScan^®^. The ^111^In-pentetreotide is an ^111^In-DTPA conjugate of octreotide that specifically binds somatostatin receptors, able to provide information regarding the presence and the amount of STRR on cell’s surface in patients with GEP-NENs. Scintigraphic imaging with ^111^In-pentetreotide shows a wide range of sensitivity (from 15 to 100%) in detecting GEP-NENs, according to tumor type, lesion size and acquisition modality [28,29,30,31,32]. Somatostatin receptor scintigraphy is limited in organs with higher physiological uptake and for detection of small lesions due to suboptimal physical resolution of the isotopes used for SPECT imaging [33]. It should be considered that SPECT/CT imaging may help localize foci of abnormal tracer uptake more accurately than planar or SPECT alone and should be considered whenever indicated and available [33,34].

During past years, PET/CT imaging after administration of somatostatin analog has been introduced for the evaluation of patients with GEP-NENs [35,36]. Integrated PET with CT in a single unit (PET/CT) provides several advantages, including a more accurate localization and characterization of detected lesions and improved imaging quality thanks to attenuation correction. Therefore, PET/CT has largely supplanted traditional SPECT imaging thanks to a higher spatial resolution, shorter acquisition times and tracer uptake quantitation.

For this purpose, somatostatin analogs are labeled with Gallium-^68^ [^68^Ga]. A germanium-68/gallium-68 generator system is used to produce ^68^Ga using solid and liquid targets. The commercial generators are characterized by a 10–100 mCi capacity and are eluted with HCl with low metal/chemical impurities. The availability of generators system allow to produce ^68^Ga also in nuclear medicine laboratories without available cyclotrons [37]. The somatostatin analogs are short peptides linked to the ^68^Ga by a bifunctional chelate (1,4,7,10-tetraazacyclododecane-1,4,7,10-tetraacetic acid-DOTA). ^68^Ga-DOTA-peptides bind to the SSTRs over-expressed on NET cells’ membrane, and the complex is then internalized. Three different ^68^Ga-DOTA- peptides are utilized: ^68^Ga-DOTA-Tyr(3)-octreotate (^68^Ga-DOTATOC), ^68^Ga-DOTA,1-Nal(3)-octreotide (^68^Ga-DOTANOC) and ^68^Ga-DOTA, D-Phe1,Tyr (3)-octreotide (^68^Ga-DOTATATE). All these agents are comparable in terms of sensitivity and specificity. ^68^Ga-DOTANOC binds SST receptors 2, 3 and 5. ^68^Ga-DOTATOC shows a good affinity for SSTR 2 and 5 and a lower affinity for SSTR3, as compared to ^68^Ga-DOTANOC. ^68^Ga-DOTATATE has a predominant high affinity for SSTR2 and low affinity for SSTR5 and SSTR3 [38,39]. It has been observed that PET/CT imaging that uses ^68^Ga-DOTA-peptides has a high sensitivity (90%, range 64–100%) and specificity (95%, range 83% to 100%) in detecting disease in patients with GEP-NENs, allowing earlier diagnosis than conventional morphological imaging modalities [35,36]. Sensitivity varies according to tumor types and grades, reflecting the density of SSTR expression on cells’ membrane. Gabriel et al. [40] described a higher detection rate performance of ^68^Ga-DOTATOC in 84 patients with NETs, as compared to traditional SPECT and CT imaging. Frilling et al. [41] demonstrated that ^68^Ga-DOTATOC PET/CT imaging showed pathologically increased uptake for at least one tumor site in 52 patients with GEP-NETs. Moreover, ^68^Ga-DOTATOC visualized the primary tumor region in 3 of 4 patients and additional hepatic and or extrahepatic metastases in 22 of 33 subjects in which disease was not identified on CT and/or MR, changing initial treatment decision in 31 (59.6%) patients. This study suggested that ^68^Ga-DOTATOC PET/CT is superior to CT and or MRI for detection and staging of NET. Prasad et al. [42] also confirmed that the ^68^Ga-DOTANOC was more helpful than CT and SPECT imaging with somatostatin analogs for the detection of unknown primary tumors in patients with confirmed NET secondary lesions. Moreover, Ambrosini et al. [43] reported a good sensitivity of ^68^Ga-DOTANOC in also detecting small lesions, in particular nodal and bone disease. These data were also confirmed by Putzer et al. [44] in 51 patients with well differentiated NET, where ^68^Ga-DOTATOC PET/CT performed better than CT and SPECT imaging with somatostatin analogs for the early detection of bone NET secondary lesions (sensitivity of 97%, specificity of 92%). The main studies exploring the diagnostic ability of ^68^Ga-DOTA-peptides PET/CT imaging in identifying GEP-NENs are reported in Table 1.

Current guidelines recommend functional imaging by using radiolabeled somatostatin analogs in order to localize primary tumors and detect sites of metastatic disease, for re-staging of patients with known disease, to monitor the effects of therapy, including surgery, radiotherapy, chemotherapy or somatostatin analog therapy [45]. Moreover, the main opportunity for ^68^Ga-DOTA-peptides imaging is the selection of patients for PRRT to obtain a prognostic parameter for response of subsequent therapy.

## 5. Imaging Analysis

Nuclear imaging by radiolabeled somatostatin analogs is able to estimate the amount of SSTR on tumor cells. In patients with GEP-NENs, the expression of SSTR might predict the efficacy for treatment with somatostatin analogs, with a significant impact on outcome. [46]. However, several pitfalls in interpreting nuclear imaging by somatostatin analogs may occur: the physiologic distribution of the tracer that includes healthy tissues and inflammatory processes that might lead to a potential false positive [47]. In particular, physiological uptake of ^68^Ga-DOTA-peptides is reported in several tissues, including spleen, adrenal glands, pituitary gland, liver, thyroid and salivary glands. Moreover, the uncinate process of the pancreas has a high density of SSTR, leading to increased activity also in absence of disease. Moreover, the tracer undergoes renal excretion where it is filtrated and partially reabsorbed in the proximal tubule. Other foci of potential pitfalls include sites of osteoblastic activity and active inflammatory processes that express SSTR2 receptors. It should also be taken into account that non-neuroendocrine tumors, including renal cells carcinoma, may express SSTR [48]

Moreover, individual tumors can have heterogeneous levels of SSTR expression related to their degree of differentiation. Therefore, several imaging biomarkers have been proposed to standardize imaging assessment and improve diagnostic accuracy, reproducibility and prognostic impact in patients with GEP-NENs.

The Krenning score was first introduced for the analysis of ^111^In-pentetreotide planar images and then was identically adapted to ^68^Ga-DOTA-peptides PET/CT imaging. It is a 4-point visual score, based on the analysis of the lesion with the highest SSTR ligand uptake at functional imaging (H-lesion): 0 no uptake; 1, very low uptake; 2, uptake less than or equal to that of the liver; 3, uptake greater than the liver; and 4, uptake greater than that of the spleen [49,50,51]. Hope et al. [51] compared the Krenning scores obtained in 150 patients with NET, who underwent both SPECT and PET/CT imaging by somatostatin analogs. They concluded that ^68^Ga-DOTA-peptides PET/CT results in higher Krenning scores than ^111^In-pentetreotide, in particular for lesions < 2 cm. Therefore, in the evaluation of SSTR density, PET/CT imaging should be preferred in patients with small lesions even if negative at traditional SPECT imaging. The Krenning score has been related to histopathological tumor grade and prognosis [50,51]. In order to standardize imaging analysis and overcome potential pitfalls in PET/CT interpretation, Werner et al. [52] proposed the SSTR-RADS, a structured 5-point score system for [^68^Ga] Ga-DOTA-peptides PET imaging adapted from the PSMA-RADS score already proposed for prostate imaging [53]. The main application seems to be the selection of patients’ candidates to PRRT.

Therapy with radiolabeled somatostatin analog should be considered in patients with an overall SSTR-RADS score of 4 (“positive uptake in site typical for NET lesions without corresponding anatomic finding”) or 5 (“intense uptake in site typical for NET with corresponding findings on conventional imaging”). Lately, volumetric parameters for functional imaging have also been evaluated. Abdulrezzak et al. [54] introduced two new volumetric parameters: the somatostatin receptor expressing tumor volume (SRETV) and the total lesion somatostatin receptor expression (TLSRE). There is still little scientific evidence about these parameters, and cut-off values related to functional volume burden and outcome have not yet been addressed.

## 6. Functional Imaging by ^18^F-FDG PET/CT

The glucose analog ^18^F-FDG is the most commonly used oncological radiotracer, for staging, re-staging and evaluation of response to therapy in several tumors [55,56,57]. Cancer cells typically show an increased glucose metabolism that facilitates rapid tumor growth, and it is related to a higher degree of malignancy [58,59]. Therefore, lesions with high ^18^F-FDG uptake are more clinically aggressive [60,61,62,63]. The ^18^F-FDG uptake might be influenced by phenotype, mitotic index and grade of the primary tumor [64]. Hybrid imaging by ^18^F-FDG PET-CT is generally recommended for evaluation of patients with GEP-NENs with high proliferation index (G3) and poorly differentiated neuroendocrine carcinomas [65,66]. In a large prospective study on 166 patients with GEP-NENs, Binderup et al. [67] reported that overall survival (OS) and progression-free survival (PFS) were significantly better for 18F-FDG–negative when compared with ^18^F-FDG–positive patients, reflecting tumor aggressiveness. It should also be considered that, differently from histological assessment of selected lesions, ^18^F-FDG PET/CT takes the advantage of allowing a whole-body evaluation of the entire disease burden. The loss of SSTR expression was found to coincide with an increase in glucose utilization in cells [66].

Therefore, the combined evaluation of PET/CT imaging by ^68^Ga-DOTA -peptides and 18F-FDG in patients with GEP-NENs has been largely investigated. Cingarlini et al. [68] evaluated 35 patients with surgically resected G1 and G2 pancreatic NETs, who underwent ^68^Ga-DOTATOC and ^18^F-FDG PET/CT imaging. In these patients, ^68^Ga-DOTATOC PET/CT showed a high sensitivity in detecting G1 (100%) and G2 (92%) pancreatic NETs while ^18^F-FDG PET/CT showed a lower sensitivity for both G1 (20%) and G2 (76%) tumors, respectively.

The combined approach helped better define the phenotype of disease, suggesting its potential role in prognostication and risk stratification of patients with pancreatic GEP-NENs. Sansovini et al. [69], in a retrospective cohort of 60 patients with pancreatic NETs treated with PRRT, reported a median PFS of 21.1 months for patients with a positive ^18^F-FDG PET/CT and a significantly longer PFS of 68.7 months for patients with negative baseline ^18^F-FDG PET/CT. Similarly, Severi et al. [70] evaluated 52 patients with progressive advanced G1 and G2 NETs treated with ^177^Lu-DOTATATE; of those, 33 had positive and 19 negative ^18^F-FDG PET/CT imaging. The disease control rate was 100% in patients with negative ^18^F-FDG and 76% in patients with positive imaging, with a PFS of 32 and 20 months, respectively. Those data confirm that ^18^F-FDG PET/CT has a high prognostic value. In well differentiated G1 and G2 NETs, negative ^18^F-FDG scans were linked to a significantly better PFS after PRRT regardless of the Ki67 grading score. On the contrary, tumors with a positive ^18^F-FDG uptake led to a shorter PFS and OS, independently from other markers of aggressiveness, such as grading and Ki67 index [61,67]. Nilica et al. [71] evaluated 66 patients with histologically proven NET, who underwent PRRT and three combined ^68^Ga-DOTATOC and ^18^F-FDG PET/CT studies. In these patients, the presence of ^18^F-FDG uptake correlated with a higher risk of progression of disease. However, the absence of ^18^F-FDG uptake at baseline evaluation does not exclude the occurrence of positive scans during follow-up. Therefore, the patients with a negative ^18^F-FDG PET/CT imaging may show positive scans during the follow-up, also in the presence of lower grades of disease.

Recently, Chan and coworkers proposed the NET-PET grade, a new score incorporating both ^68^Ga-DOTA-peptides and ^18^F-FDG PET/CT imaging findings, that well correlates with outcome [72,73]. 

The ^18^F-FDG PET/CT has been shown to be useful in GEP-NENs patients with G2 G3 proliferation index, in particular in those with rapidly progressive disease and in patients positive to CT and/or MRI and negative to imaging with DOTA-peptides. However, ^18^F-FDG PET/CT should be considered not as a competitor but as a complementary tool to ^68^Ga-DOTA-peptides imaging. The identification of lesions showing areas of match or mismatch at metabolic and receptorial imaging (flip-flop phenomenon) [66] is a powerful tool in prognostic stratification of GEP-NENs patients. 

**Figure 2:**
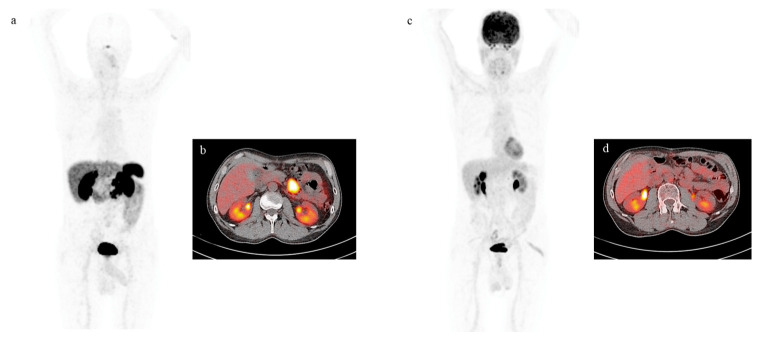
MIP views and transaxial fusion images of ^68^Ga-DOTATOC PET/CT (**a**,**b**) and of 18F-FDG PET/CT (**c**,**d**) scans performed on the same patient with metastatic mesenterial lymph nodes from ileal NET previously removed. Focal uptake is clearly visible on 68Ga-DOTATOC PET/CT images (**a**,**b**) while it is absent on ^18^F-FDG PET/CT images (**c**,**d**).

**Figure 3:**
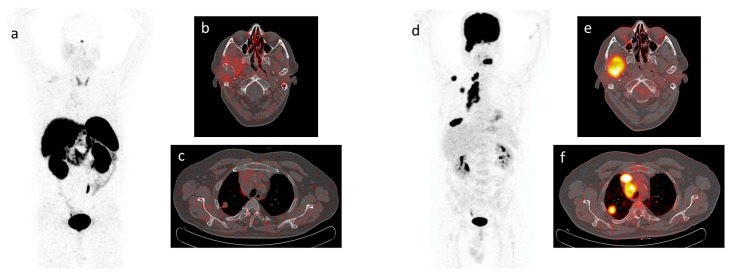
MIP views and transaxial fusion images of ^68^Ga-DOTATOC PET/CT (**a**–**c**) and of ^18^F-FDG PET/CT (**d**–**f**) scans performed on the same patient with metastatic NET of the lung. Focal uptake is absent on ^68^Ga-DOTATOC PET/CT images (**a**–**c**) while on ^18^F-FDG PET/CT images focal uptake is clearly visible on the right mandible (**e**), primary tumor and mediastinal lymph nodes (**f**).

Two representative cases of patients who performed both ^68^Ga-DOTATOC PET/CT and ^18^F-FDG PET/CT scans are depicted in Figure 2 and Figure 3, respectively.

## 7. PRRT by Radiolabeled Somatostatin Analogs

In GEP-NENs patients with high SSTR expression and advanced disease, PRRT has been demonstrated to be an effective systemic treatment. 

The PRRT protocols consist on the systemic administration of a radiopharmaceutical composed of a β-emitting radionuclide, chelated to a specific somatostatin analog. The two most used radionuclides are Yttrium-90 (^90^Y) and Lutetium-177 (^177^Lu), which show substantial differences in terms of decay energy and penetration depth. In particular, ^90^Y is a pure β-emitting isotope; it decays with an energy of 2.27 MeV and an average penetration depth of 11 m. Differently, ^177^Lu emits β-particles with a lower energy (0.49 MeV) and a shorter penetration range (2 mm). In addition, ^177^Lu allows the performance of post-treatment imaging thanks to its ability of emitting γ photons, with an energy of 113 keV and 208 keV. Despite multiple therapeutic protocols that have been proposed during the last years, the recommended activities range from 1.8 to 2.5 GBq for ^90^Y-DOTATOC (time interval of 8–10 weeks, for 4 cycles) and from 3.7 to 7.4 for ^177^Lu-DOTATATE (time interval of 6–12 weeks, for 4 to 5 cycles) [68]. The clinical effect of ^90^Y-DOTATOC has been previously evaluated in 90 symptomatic patients with carcinoid tumors with at least one symptom refractory to octreotide and one measurable lesion [74]. The patients were treated with 3 cycles at 6-week intervals. According to Southwest Oncology Group (SWOG) criteria [75], defined to assess tumor response to treatment and standardized grades for evaluation of treatment toxicity, 67 patients (74%) showed stable disease or response to therapy, with a significant trend in improvement of symptoms. Mean PFS was significantly longer for the 38 patients who showed improvement of symptoms as compared to 18 patients who did not (18.2 vs. 7.9 months). The treatment resulted in being well tolerated; only two cases of severe but reversible renal toxicity were registered. It should be noted that ^90^Y-DOTATOC takes advantage of a higher β particle emission as compared to ^177^Lu-DOTATATE; however, ^177^Lu-DOTATATE shows higher SSTR2 affinity, a longer residence time in tumor and a lower kidney exposure. Moreover, i ^177^Lu has a double emission that allows performing a post-therapy scan in order to assess tracer distribution and to perform dosimetry evaluations.

**Figure 4:**
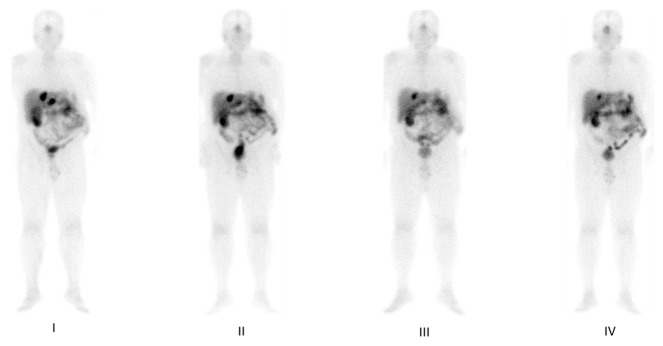
The post-therapy whole body scan performed after **I**, **II**, **III** and **IV** cycle of therapy with ^177^Lu-DOTATATE in a patient with metastatic pancreatic NET, shows a focal and progressive reduction of uptake in the liver and lymph node metastases.

**Figure 5:**
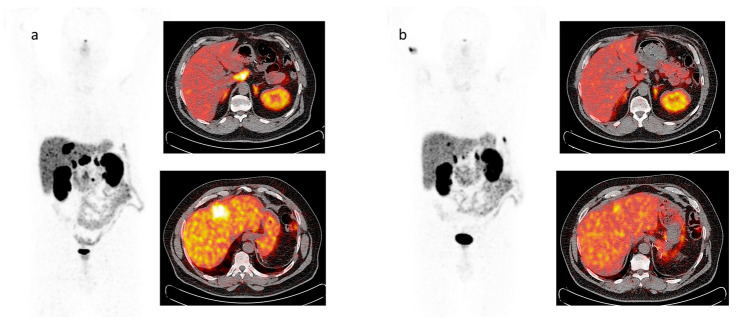
MIP views and transaxial fusion images of ^68^Ga-DOTATOC PET/CT scans performed before (**a**) and after (**b**) therapy with ^177^Lu-DOTATATE in a patient with metastases to the liver and abdominal lymph nodes from pancreatic NET previously removed. Focal uptake on liver and lymph node metastases is reduced on the post-therapy images.

The post-therapy whole body and ^68^Ga-DOTATOC PET/CT scans performed before (a) and after (b) PRRT in the same patient affected by pancreatic NET with liver and nodal metastases are shown in Figure 4 and Figure 5. In both imaging methods, areas of focal uptake on liver and lymph node are reduced on the post-therapy images.

The efficacy and safety of ^177^Lu-DOTATATE were first evaluated by Kwekkeboom et al. [76] in 310 and 504 patients, respectively. All patients were treated with a cumulative activity of 27.8–29.6 GBq, administered at intervals of 6–10 weeks for 4 cycles. Complete response (CR) and partial response (PR) were observed in 2% and 28% of 310 patients with GEP-NET, respectively. Moreover, PFS was 40 months, with a survival benefit of 40 to 72 months from diagnosis. The treatment resulted as safe and well tolerated, with a low incidence of grade 3 or 4 subacute hematologic toxicity (3.6% of administrations); myelodysplastic syndrome occurred in only 3 patients and temporary liver toxicity in 2 patients. Subsequent evidence [77,78,79,80] confirmed that ^177^Lu-DOTATATE shows a very good tolerability, with minimal toxicity to kidney and bone marrow.

The NETTER-1 is a pivotal phase III randomized trial, where the safety and efficacy of ^177^Lu-DOTATATE has been tested in 229 patients with GEP-NET in disease progression during and after SSA therapy [81]. According to the study protocol, patients were randomized and assigned to receive high doses of octreotide alone every 28 days, or 4 cycles of 7.4 GB of ^177^Lu-DOTATATE at 8-week intervals, associated with octreotide. In the first interim analyses, the investigators observed that PFS resulted in being significantly higher in patients treated with PRRT (65.2%) as compared to those who received octreotide alone (10.8%). Moreover, the ^177^Lu-DOTATATE group had a higher rate of positive response to therapy versus the control group (18% vs. 3%). The OS, evaluated at the time of interim analysis, registered 14 deaths in the ^177^Lu-DOTATATE group versus 26 in the control group. The NETTER-1 trial confirmed the good tolerability of the treatment; grade 3 or 4 neutropenia, thrombocytopenia and lymphopenia occurred in 1%, 2% and 9% of the PRRT patients, respectively. According to these preliminary results, ^177^Lu-DOTATATE has been approved as a therapeutic option for patients with progressive, advanced, well-differentiated G1 and G2 GEP-NETs. Final OS overall survival and long-term safety results from NETTER-1 have been recently published [82]. In this important study, the final analysis occurred 5 years after the last patient of 231 subjects was randomly assigned. The median OS of the ¹⁷⁷Lu-DOTATATE group (48.0 months) did not result in significant improvement as compared to the control group (36.3 months), but the difference in OS between the two study groups of 11.7 months should still be considered in practical and clinical terms. Moreover, it should be highlighted that 33% of patients in the high dose octreotide group crossed over the PRRT group.

In a subsequent prospective phase II study by Sansovini et al. [83], 52 consecutive patients with advanced G1-G2 pancreatic NETs were treated with 5 cycles every 6–8 weeks. According to kidney and bone marrow parameters, two different cumulative activities were administered; in particular, 26 patients received a mean full activity (FA) of 25.5 GBq, and 26 received a mean reduced activity (RA) of 17.8 GBq. The disease control rate, defined as the sum of CR + partial response (PR) + stable disease (SD), was considered as endpoint, and it was observed in 85% of the FA patients and in 77% of the RA group. The median PFS was not reached in the FA group and was 20 months in the RA group, without significant differences in OS s between the two groups. In conclusion, ^177^Lu-DOTATATE therapy showed antitumor activity even in patients with RA dose. However, PFS was significantly longer after a total activity of 27.8 GBq, suggesting the use of this assay in well selected patients.

In a more recent paper, the same group [84] published the results of a 10-year follow-up of a phase II trial conducted in 43 patients with progressive metastatic GEP-NETs. All patients had positive PET or SPECT imaging with somatostatin analogs in known lesions, and they were monitored for a median period of 118 months (range 12.6–139.6). Median PFS in patients receiving a lower activity of 18.5 GBq was identical to that of patients treated with 27.5 GBq. The median OS was 71.0 months in the group who received a lower activity and 97.6 months in the other group. Age over 65 years at the time of PRRT was also significant for OS, and of note, no late hematological or renal toxicity was observed in either group. In conclusion, ^177^Lu-DOTATATE became “de facto” the more widely used agent for PRRT thanks to its tolerability and efficacy.

The more relevant studies exploring the efficacy of ¹⁷⁷Lu-DOTATATE PRRT are summarized in Table 2. The results of these studies could be a starting point in the future to be able to conduct any studies focused on a greater dose fractionation linked to a lower dose toxicity.

In order to optimize treatment protocols, a personalized dosimetric approach has been proposed in patients with GEP-NENs candidates to PRRT administration. 

The personalized dosimetry aims to provide a sufficient absorbed dose to the target lesions, with a concomitant reduction in the absorbed dose to vulnerable organs [85,86,87]. It should be considered that the kidneys and bone marrow are organs at risk, where the maximum tolerated doses are 23 Gy and 2 Gy, respectively [85,86,87]. Ilan et al. [88] calculated the tumor-absorbed dose for 24 metastases in 24 patients with NETs, using sequential SPECT/CT post-therapy acquisitions, 24, 96 and 168 h after ^177^Lu-DOTATATE PRRT infusion. The metastatic lesions receiving higher absorbed doses appeared more likely to respond to PRRT in terms of tumor size reduction [88]. Del Prete et al. [89] estimated a lesion absorbed dose > 130 Gy as a cut-off to obtain a significant reduction in tumor size [89]. 

Several authors focused their attentions on the optimal timing for SPECT/CT scans: Simplified protocols have been proposed in order to reduce the number of acquisitions and have made dosimetry more accessible [85,87,90]. 

Following the tendencies of combined treatments in oncology, the possibility of associating PRRT to chemotherapeutic agents has been investigated, to increase the therapeutic response and to prolong the PFS. Capecitabine, the oral prodrug of 5-fluorouracile (5-FU may act as a radiosensitizer in synergy with radionuclide therapy, and this could be particularly useful in aggressive or radioresistant disease. Several single-arm phase II trials have investigated the tolerability and efficacy of combining ^177^Lu-DOTATATE with capecitabine using various administration schedules [91,92,93]. In a prospective phase II study, Nicolini et al. [94] aimed to test the efficacy and toxicity of ^177^Lu-DOTATATE associated with metronomic capecitabine in 37 GEP-NETs patients with previous positive ^18^F-FDG PET/CT imaging. The occurrence of grade 3 or 4 hematological toxicity was observed in 16.2% of patients, while no patients had renal toxicity for the entire follow-up. Moreover, response to therapy was assessed in 33 patients; of those, 10 (30%) had PR, and 18 (55%) had SD, during a median follow-up of 38 months. The authors concluded that combining ^177^Lu-DOTATATE and capecitabine is active and well tolerated, and this could be useful in patients with ^18^F-FDG positive GEP-NETs. Although these preliminary results are encouraging, available data are limited to propose this combined protocol in the treatment of GEP-NENs patients; for this purpose, robust randomized phase III studies are needed.

## 8. Response to Therapy: Which Criteria?

In order to evaluate the response to therapy in patients with oncological disease, reproducible and standardized quantitative response criteria are needed. For this purpose, the World Health Organization first introduced the concept of “objective response criteria”, followed in 1992 by the new SWOG response criteria [6,75,84].

In order to simplify the evaluation of response to therapy and reduce error associated with WHO and SWOG criteria, the RECIST criteria were first introduced and then modified as RECIST version 1.1 to improve their use in clinical practice [6,75,95,96]. RECIST are based on the identification of target lesions on conventional imaging, such as CT or MR, and evaluation of the longest diameter of each target lesion. Although the RECIST 1.1 criteria are considered the gold standard in the assessment of response to treatment, some limitations have been observed, in particular regarding irregular lesions, peritoneal carcinomatosis and small lesions less than 1 cm that are particularly relevant in GEP-NENs patients. Van Vliet et al. [97] examined OS and PFS in a cohort of 268 patients with GEP and thoracic NENs treated with PRRT, according to different criteria including RECIST 1.1, SWOG and modified RECIST, where a minor response is defined by a decrease by 13–30%, and modified SWOG, where a minor response is defined by a decrease by 25–50%. No significant differences were found in both median PFS and OS between the four groups. Therefore, they concluded that both modified RECIST and SWOG criteria do not improve the accuracy of PRRT response in NETs while RECIST 1.1 and SWOG criteria seem to be comparable.

In a recent retrospective study by Huizing et al. [98] including 44 patients with NETs, images acquired prior, 3 and 9 months after PRRT were evaluated using RECIST 1.1 and Choi criteria. Choi criteria have been first proposed for gastrointestinal stromal tumors (GIST) to consider variations of density of target lesions [99]. Among a total of 110 lesions, the evaluation of PRRT response by Choi criteria, as compared to RECIST 1.1, led to a longer mean OS analysis in patients who had response to therapy but similar results in both stable disease and progressive disease group. These findings indicate that Choi criteria may identify responders more accurately.

In order to identify lesion properties more predictive of PRRT outcome, rating scales have also been proposed for metabolic imaging. In 1999, the European Organization for Research and Treatment of Cancer (EORTC) introduced the first PET scoring system, based on the evaluation of semiquantitative PET parameters, such as SUV. The PET Response Criteria in Solid Tumors (PERCIST) were further introduced based on several quantitative parameters, such as metabolic tumor volume (MTV), a measurement of tumor volume burden and total lesion glycolysis (TLG), that is the product of MTV and the SUV mean parameter [100].

Several authors focused their attention on the identification of SUV max cut-off values able to predict PRRT outcome [101,102]. Öksüz et al. [101] identified a SUV max > 17.9 on ^68^Ga-DOTATOC PET/CT as a favorable cut-off for predicting prognosis in patients who underwent PRRT. On the contrary, Gabriel et al. [102] did not find any benefit of SUV max analysis on ^68^Ga-DOTATOC PET/CT for the prediction of individual therapy response. 

Sharma et al. [103] aimed to evaluate response to therapy in 55 patients with metastatic NETs treated with PRRT, according to the following parameters: single lesion SUV max, the tumor to spleen uptake (T/S) and tumor to liver uptake (T/L) ratios and average SUV max, defined as the average SUV max of up to five target lesions in multiple organs sites. All these parameters were evaluated at baseline, follow-up and end-treatment PET/CT scan. The authors observed that only a baseline single lesion SUV max > 13.0 and an average SUV max > 10.2 were predicted of both response to therapy and PFS. It should be considered that several factors, including camera and acquisition times, may affect SUV max quantification; therefore, a common threshold value should be carefully interpreted.

Tumor heterogeneity of SSTR2 expression can be observed within a lesion or among different lesions in the same patients. It has been hypothesized that SSTR2 heterogeneity may affect clinical outcome and play an important role in predicting tumor response to therapy. Fonti et al. [104] investigated the ability of coefficient of variation (CoV), derived from PET/CT with ^68^Ga-peptides, in evaluating the heterogeneity of SSTR2 expression in 38 patients with NETs. They found that CoV values, reflecting tumor heterogeneity, vary with the type and site of malignant lesions. In particular, the higher CoV values were observed for bone lesions as compared to primary tumor and liver metastases. Some authors [54,105,106] have considered new parameters, such as the somatostatin receptor expressing tumor volume (SRETV) and the total lesion somatostatin receptor expression (TLSRE), as an expression of volumetric tumor burden of GEP NENs, but we are still far from using these parameters in daily clinical practice.

Radiomics techniques have been also proposed as a promising tool for the evaluation of SSTR2 heterogeneity. Radiomics is an emerging method able to extract innumerable features from medical images by the relationship between the intensity and position of each voxel. The potential applications of radiomics features in patients with GEP-NENs have been investigated for several purposes, including diagnosis, response assessment and prediction of long-term outcome [107]. Blazevic et al. [108] identified 68 patients with GEP-NENs at high risk to develop metastatic mesenteric masses. Through the extraction of particular CT features, the same predictive value of a multidisciplinary tumor board has been reached. Werner et al. [109] evaluated the prognostic ability of different textural features derived by PET/CT images in patients with GEP-NENS, and they found that the entropy predicted both PFS and OS. 

Despite promising results, radiomics is a still emerging method that needs far more robust and accurate data before being introduced into clinical practice.

## 9. New Advances and Future Prospectives

The current evidence confirms that available diagnostic and therapeutic radiotracers play an important role in the management of patients with GEP-NENs. However, to overcome some limitation related to tracer distribution and to improve diagnostic and therapeutic applications, new radiotracers have been proposed.

For PET/CT imaging, the availability of SST analogs labeled with ^18^F may help in overcoming several practical and economic challenges. Pauwels et al. [110] evaluated safety, dosimetry, biodistribution, pharmacokinetics and lesion targeting of ^18^F-AlF-NOTA-octreotide in comparison to ^68^Ga-DOTATATE in six healthy volunteers and six NET patients. The physiological uptake pattern was similar for both tracers; however, ^18^F-AlF-NOTA-octreotide showed a lower uptake in tumor lesions but increasing over time. Moreover, ^18^F-AlF-NOTA-octreotide takes the advantage of a lower liver/background uptake as compared to ^68^GaDOTATATE PET/CT, which allowed it to more accurately identify liver lesions. However, in patients with a high number of metastases, ^18^F-AlF-NOTA-octreotide missed more bone lesion than ^68^GaDOTATATE PET/CT. Overall, these preliminary data indicate ^18^F-AlF-NOTA-octreotide as a promising tracer for NET imaging.

In another retrospective study, Ilhan et al. [111] compared ^18^F-SiFAlin-TATE with ^68^Ga-DOTATOC in 13 NENs patients. A significantly higher ^18^F-SiFAlin-TATE uptake was described in kidneys. Moreover, among 109 lesions, tumor uptake was found to be significantly higher for ^18^F-SiFAlin-TATE in all tumor sites, with the exception of lung lesions. These preliminary results were affected by the high heterogeneity of enrolled patients; they suggest the potential utility of ^18^F-SiFAlin-TATE.

The safety and diagnostic ability of Copper-^64^ (^64^CU) MeCOSar-Tyr3-octreotate (SARTATE) were first tested by Hicks et al. [112] in 10 G1 or G2 NENs patients with positive ^68^Ga-DOTATATE-PET/CT imaging. It should be highlighted that ^64^Cu may provide several advantages in terms of improved imaging quality thanks to its attractive physical characteristics, and in the field of theragnostic, it can be used as a diagnostic partner for the therapeutic radionuclide ^67^Cu. The images acquired 1 h after ^64^Cu-SARTATE injection resulted in being comparable to those obtained by ^68^Ga-DOTATATE. Interestingly, lesion-to-liver ratio increased progressively between 4 and 24 h, improving the identification of livers metastases. All these evidences make ^64^Cu-SARTATE a safe PET/CT radiotracer, with good qualities for diagnostic studies and for prospective dosimetry for ^67^Cu-PRRT.

The fibroblast activation protein inhibitor (FAPI) is overexpressed by fibroblasts and associated with cancer and poor prognosis. It has been observed that FAPI is overexpressed by different tumors, in particular epithelial carcinomas [113]. Recently, ^68^Ga-FAPI emerged as a promising radiotracer for PET/CT imaging, and several case reports indicated substantial uptake in NENs [112].

During the last years, simultaneous PET/MRI has been introduced as a promising imaging method in the evaluation of several tumors, including GEP-NENs. Beiderwellen et al. [114] investigated the potential role of ^68^Ga-DOTA-peptide PET/MRI in eight patients with GEP-NET. In this case, PET/MRI has proved to be a promising method as regards abdominal lesions, which represent the most frequent location of the disease in GEP-NETs. Some limitations related to the use of MRI include lung and hypersclerotic bone lesions. Hope et al. [115] also showed that the simultaneous use of both ^68^Ga-DOTA-TOC and gadoxetate disodium in PET/MRI had an higher diagnostic accuracy in detecting hepatic lesions, and it was successful in whole body staging However, it should be considered that high costs and long-time acquisition may limit the wide spread of PET/MRI into clinical practice.

For therapeutic purposes, new tracers able to emit α particle have been proposed. Differently from β-emitters, α particles are able to release a higher energy in a shorter space, leading to a more accurate and localized therapeutic effect and a relative preservation of healthy tissues. Previously, α emitters have been already proposed for the treatment of patients with castration-resistant prostate cancer and bone metastatic lesions [116]. In patients with NENs, SST analogs labeled with actinium-225 (^225^Ac) or bismuth-213 (^213^Bi) have been also applied in clinical trials; however, more data are needed, and we are still clearly far from including them in clinical practice [117,118]. We hope in the progress!

## 10. Conclusions

Recent advances in functional imaging and radiometabolic therapy improved diagnosis and management of patients with GEP-NENs. The theragnostic approach, able to combine diagnosis and therapy, represent an emerging opportunity for the management of patients with GEP-NENs.

## Figures and Tables

**Table 1:** Diagnostic ability of PET/CT with ^68^Ga-DOTA-peptides in identifying GEP-NENs.

Authors	Patients (*n*)	Primary Tumor Location	Endpoints	Sensitivity
Gabriel et al. [40]	84	20 (24%) pancreas 30 (36%) GI tract 34 (41%) other sites	Identification of primary tumor and metastatic disease Comparison with SPECT and CT	97%
Ambrosini et al. [43]	223	64 (29%) pancreas 55 (25%) GI tract 104 (47%) other sites	Identification of bone metastases Comparison with CT	100%
Frilling et al. [41]	52	27 (52%) pancreas 19 (37%) GI tract 5 (10%) other sites	Identification of primary tumor and metastatic disease Comparison with CT and MRI	100%
Prasad et al. [42]	59	16 (27%) pancreas 16 (27%) GI tract 27 (46%) other sites	Identification of undiagnosed primary tumor	59%
Putzer et al. [44]	51	11 (22%) pancreas 24 (47%) GI tract 16 (31%) other sites	Identification of bone metastases Comparison with CT and bone scintigraphy	97%

GI, gastrointestinal tract.

**Table 2:** Safety and efficacy of ¹⁷⁷Lu-DOTATATE PRRT.

Authors	Patients (*n*)	Study Population	Cycles/Intervals (*n*/weeks)	Activity (GBq)	Endpoints	Median Follow-Up (Months)	PFS (Months)
Kwekkeboom et al. [71]	504	Suspected or histologically proven GEP-NETs	4/6–10	27.8–29.6 (Cumulative)	Safety, OR, OS	19	32
Strosberg et al. [76]	229	Histologically proven advanced midgut-NETs	4/8	7.4	Safety, OR, PFS	14	30
Strosberg et al. [77]	231	Histologically proven advanced midgut-NETs	4/8	7.4	OS	76	30
Sansovini et al. [78]	52	Histologically proven pancreatic NETs	5/6–8	17.8 or 25.5 (Cumulative)	Safety, OR, OS	29	29
Paganelli et al. [79]	43	Histologically proven GI-NETs	6–8	18.4 or 25.7 (Cumulative)	Safety, OR, PFS	118	59.8

NET, neuroendocrine tumors; GI, gastrointestinal tract; OR objective response; OS, overall survival; PFS, progression-free survival.

## Data Availability

No new data were created or analyzed in this study. Data sharing is not applicable to this article.
